# Socioeconomic Inequalities in Chronic Disease in Kharameh Cohort Study: A Population‐Based Cross‐ Sectional Study in Southern Iran

**DOI:** 10.34172/aim.2023.03

**Published:** 2023-01-01

**Authors:** Leila Moftakhar, Masoumeh Ghoddusi Johari, Abbas Rezaianzadeh

**Affiliations:** ^1^Student Research Committee, Shiraz University of Medical Sciences, Shiraz, Iran; ^2^Breast Diseases Research Center, Shiraz University of Medical Science, Shiraz, Iran; ^3^Colorectal Research Center, Shiraz University of Medical Science, Shiraz, Iran

**Keywords:** Concentration index, Inequality, Iran, Non-communicable diseases, PERSIAN Cohort

## Abstract

**Background:**

The trend of chronic diseases is increasing globally. Socioeconomic status (SES) is a major factor underlying many chronic diseases. This study was conducted to investigate the socioeconomic inequalities in distribution of chronic diseases in Iran, as a middle-income country.

**Methods:**

This cross-sectional study was conducted using the baseline data of the Kharameh cohort study, that were collected between 2014 and 2016. The number of participants in this study was 10663 people in the age range of 35 to 70 years. Principal component analysis was used for calculating the SES of the people under study. In addition, we used concentration index and concentration curve to measure socioeconomic inequality in chronic disease.

**Results:**

The mean age of 10,663 participants in our study was 52.15±8.22 years and the male to female ratio was 1.26. Recurrent headache (25.8%( and hypertension (23.5%) were the most prevalent diseases. The concentration index showed that the distribution of movement disorder, recurrent headaches and gastroesophageal reflux diseases is significantly concentrated among people with low SES, and obesity among people with high SES. The results of the analysis by gender were similar to the results seen in all participants.

**Conclusion:**

The findings of this study show that socioeconomic inequality is the cause of the concentration of non-communicable diseases among people with low socio-economic status. Therefore, health policy makers should pay special attention to identifying vulnerable subgroups and formulate strategic plans to reduce inequalities.

## Introduction

 Non-communicable diseases are one of the most important public health challenges.^1-3^ Theprolonged period of the disease, slow progression, and lack of transmission to others are characteristics of non-communicable diseases. The four main types of chronic diseases are cardiovascular disease, diabetes, chronic respiratory disease and cancer.^4^ Although preventable, they are the main cause of morbidity, mortality and disability in the world.^5^ According to reports of the World Health Organization, non-communicable disease will be the leading cause of death in 2030, and the number of deaths from them will increase from 38 million in 2012 to 52 million in 2030.^2,3^ Non-communicable diseases are the cause of about 68% of deaths worldwide, 80% of which occur in low- and middle-income countries.^6-8^ Chronic diseases reduce the quality of life, create higher levels of disability and cause problems in performing daily activities.^6^

 Numerous studies have shown that many risk factors play important roles in the development of chronic diseases, including smoking, alcohol consumption, inadequate consumption of fruits and vegetables, lack of physical activity,^9^ hypertension, overweight and obesity, hyperlipidemia and a family history of chronic disease.^2,10,11^ In addition, the socioeconomic status (SES) of individuals is an important determinant of health inequalities.^1,2,5,7,12^ Health inequality is defined as differences in health status in a certain population group.^1^ This issue is usually due to inequality in the SES of individuals. Some of the factors that cause inequality in the SES are ethnicity, education, and income.^13^

 The effect of SES inequality on chronic diseases has been studied in many countries,^5^ and the results show that there is a strong correlation between SES, with people with low SES having a higher prevalence of risk factors for chronic diseases.^2,5,14^ Several studies from high-income countries have also shown that the rate of chronic diseases is higher in marginalized and deprived people than that of the people in higher levels.^3^ But there is little evidence that the distribution of SES is a risk factor for chronic diseases in low- and middle-income countries.^15^

 Due to the increasing trend of chronic diseases, it is recommended to develop preventive and effective methods and strategies to reduce their heavy economic burden.^16^ Despite advances in health indicators in recent years, health inequalities remain a global challenge within and across regions and countries.^17^ As a result, it is necessary to have a clear picture of SES inequalities in non-communicable diseases to create more effective policies and programs.However, few studies have been done in this regard in different regions of Iran for this purpose. Therefore, this study was conducted to determine SES inequalities in non-communicable diseases in Kharameh city in Iran.

## Materials and Methods

###  Study Setting

 This study was performed on 10 663 people aged 35 to 70 years in the year 2020 using baseline data of Kharameh cohort study, which was started in 2014 in the Fars province. Kharameh cohort is part of a cohort research in Iran (PERSIAN Cohort) which is a population-based study.The participation rate of this group of people in the study was 97.3%.

###  Study Design

 This is a cross-sectional study.

###  Study Procedure

 To start the study, first the trained personnel referred to the homes of all residents of Kharameh city to identify all people aged 35 to 70 years and register their names.Then, by providing the necessary explanations about the purpose and manner of conducting the Kharameh cohort study, these individuals were invited to participate in the study. After their visit to Kharameh cohort center and obtaining informed consent from them to participate in the study, each person was assigned a special barcode that was used in all stages of the study.

###  Data Collection

 To gather information, all participants in the study were interviewed face to face by nutrition experts, physicians and trained interviewer staff, and their information was collected in the relevant questionnaires and recorded electronically online. The questionnaires of this study consisted of three sections: general, medical and nutritional, which included 482 questions. Demographic information, disease status, SES, lifestyle, and behavioral factors are collected during the interview. The medical information of the individuals, including any disease that was recorded during their self-declaration, was reviewed by two physicians trained in this study, and all their medical records were reviewed. The anthropometric status of individuals was measured and recorded according to the defined standards. Other details of the cohort study can be found elsewhere.^16^

 The required information for this study consisted of two parts: first, information related to chronic diseases including diabetes (fasting blood sugar ≥ 126),^18^ hypertension (systolic blood pressure ≥ 140 mm Hg and/or diastolic blood pressure ≥ 90 mm Hg),^19^ chronic lung disease, depression, learning disability, gastroesophageal reflux, obesity (body mass index over 30),^20^ asthma, osteoporosis, movement disorder and recurrent headaches.

 The second part contains information that helps determine the SES of individuals. This information consists of the type of home ownership (personal or rental), area of the house, number of rooms in the house, having a landline, owning a washing machine, having a dishwasher, havinga color TV (regular or plasma), having a separate freezer, having a vacuum cleaner, having a computer or a laptop, access to the internet at home, access to a bathroom and toilet at home or in the yard, and having a car and its price.

 Study outcome: The final outcome of this study is to investigate the effect of SES inequality on non-communicable diseases.

###  Statistical Analysis

 The wealth index score was used to measure the SES of families. This index has fewer fluctuations than measurements based on income. We used principal component analysis to calculate people’s asset index. To perform this analysis, all variables related to measuring the level of SES were included in the analysis. In this analysis, qualitative variables were considered as numeric variable and entered into the analysis along with all quantitative variables. Finally, the results of principal component analysis gave us a numerical value as an indicator of SES class of each individual. Then, we divided the obtained asset index into 5 categories based on the percentile (very low, low, medium level, high and very high).

 Finally, we used the concentration index for the calculation of SES inequalities in non-communicable diseases. The concentration index is used as a tool to quantify the degree of wealth inequality in a health variable, and originates from the concentration curve which is shown by a line between + 1 to -1 in curve.^21^ In this curve, the x-axis shows the cumulative percentage of the population under study who were ranked based on the SES status, and on the y-axis, the cumulative percentage of the health variable (which is chronic diseases in this study) is shown.

 If the distribution of health status is equal in all aspects of the society, the concentration curve will be diagonal and will overlap with the 45-degree equalization line (the value of the concentration index is zero). If the health status is concentrated in the deprived classes of the society, the concentration curve will be at the top of the diagonal line (the value of the concentration index is negative). If the concentration curve is below the equality line, the unfavorable status of health is concentrated among the high class of society. (The value of the concentration index is positive).

 Finally, for diseases that had SES inequality (dependent variable), a separate logistic regression analysis was performed to investigate the effect of SES class on the chance of that disease. The effect of age and sex was also adjusted to control the confounders. A significance level of 0.05% was considered. Statistical analyses were performed in the Stata software version 13.

## Results

 The mean age of individuals under study was 52.15 ± 8.22 years. Participants in the study were 44.2% female and 55.8% male. The demographic characteristics of individuals based on their SES are reported in Table 1.

**Table 1:** Descriptive Characteristics of Participants by Socioeconomic Status in Kharameh, Southwestern Iran, 2020

**Variable**	**Class**	**Very Low** **No. (%)**	**Low** **No. (%)**	**Medium** **No. (%)**	**High** **No. (%)**	**Very High** **No. (%)**	**Total Number**
Gender	Female	1501 (25.2)	1299 (21.8)	1164 (19.5)	1054 (17.7)	925 (15.5)	5943
	Male	632 (133)	833 (17.6)	969 (20.5)	1078 (22.8)	1207 (25.5)	4719
Age	30-50	878 (18.4)	885 (18.5)	891 (18.6)	1014 (21.2)	1102 (23.1)	4770
	50-75	1255 (21.3)	1247 (21.1)	1242 (21.8)	1118 (19)	1030 (17.5)	5892
Education	Illiterate	1841 (33)	1507 (26.9)	1163 (20.8)	782 (14)	293 (5.2)	5586
	Elementary	224 (8.3)	438 (16.3)	635 (23.7)	723 (27)	656 (24.5)	2676
	Middle school	49 (44.3)	132 (11.6)	192 (16.9)	328 (28.8)	435 (38.2)	1136
	High school	13 (1.8)	43 (6.1)	108 (15.3)	199 (28.3)	339 (48.2)	702
	University	6 (1.07)	12 (2.1)	35 (6.2)	100 (17.7)	409 (72.7)	562
Residence	Urban	194 (5.06)	418 (10.9)	670 (17.5)	1064 (27.7)	1485 (38.7)	3831
	Rural	1939 (28.3)	1714 (25.09)	1463 (21.4)	1068 (15.6)	647 (9.4)	6831
Job*	No	1162 (22.5)	1080 (20.9)	10277 (19.9)	992 (19.2)	885 (17.2)	5146
	Yes	971 (17.6)	1052 (19.07)	1106 (20.05)	1140 (20.6)	1247 (22.6)	5516
BMI**	Under weight	121 (29.2)	110 (26.5)	74 (17.8)	58 (14)	51 (12.3)	414
	Normal	891 (22.9)	818 (21)	771 (19.8)	736 (18.9)	662 (17)	3878
	Overweight	359 (18.7)	847 (19)	897 (20.1)	944 (20.1)	1001 (22.4)	4450
	Obese	2132 (20)	356 (18.5)	391 (20.4)	394 (20.5)	416 (21.7)	1916

*Job, The employment status of the individual at the time of participation in the study; **BMI (body mass index), Underweight (BMI ≤ 18.49), Normal (BMI: 18.5 to 24.9), Overweight (BMI: 25 to 29.9), Obesity (BMI ≤ 30).

 Among the diseases studied, recurrent headache (25.8%, 24.9–26.6) and hypertension (23.5%, 22.7–24.3) were the most prevalent. Also, the prevalence of other diseases in the population under our study was as follows: diabetes (14.9%, 14.2–15.6), obesity (17.9%, 17.2–18.7) and gastroesophageal reflux disease (7.6%, 7.2–8.2). The prevalence of depression, asthma and movement disorders was almost the same in our study (5.3% to 5.6%). The prevalence of other diseases in the population under study is reported in Table 2.

**Table 2:** Prevalence of Non-communicable Diseases in Kharameh, Southwestern Iran, 2020

**Disease**	**Total Number of Patients**	**Prevalence%**	**95% CI**	
			**Upper**	**Lower**
Diabetes*	1593	14.9	14.2	15.6
Hypertension*	2510	23.5	22.7	24.3
Chronic Lung Disease	294	2.7	2.4	3
Depression	569	5.3	4.9	5.7
Learning Disability	25	0.2	0.1	0.3
Gastroesophageal Reflux	821	7.6	72	82
Obesity*	1916	17.9	17.2	18.7
Asthma	576	5.4	4.9	5.8
Movement Disorder	599	5.6	5.1	6
Recurrent Headaches	2725	25.8	24.9	26.6

*Diabetes (Fasting blood sugar ≥ 126), Obesity (body mass index over 30), Hypertension (systolic blood pressure ≥ 140 mm Hg and/or diastolic blood pressure ≥ 90 mm Hg).

 The normalized concentration index was used to estimate the SES inequality in the distribution of chronic diseases across the total population and in men and women separately. The results of this analysis showed that the concentration index of movement disorder, recurrent headaches and gastroesophageal reflux was -0.15, -0.025 and -0.07, respectively, which indicates that the distribution of diseases is significantly concentrated among people with a low SES. Also, concentration index was 0.114 for obesity, which shows a significant concentration of obesity among people with high SES (Table 3).

**Table 3:** Normalized Concentration Index in Non-communicable Diseases by Sex in Kharameh, Southwestern Iran, 2020

**Disease**	**Female**		**Male**		**Population**	
	**Concentration Index**	**95% CI**	**Concentration Index**	**95% CI**	**Concentration Index**	**95% CI**
Diabetes*	0.003	(-0.000–0.006)	-0.003	(-0.000–0.006)	-0.002	(-0.004– -0.000)
Hypertension*	0.002	(-0.001–0.006)	-0.001	(-0.004–0.001)	-0.004	(-0.007– -0.001)
Chronic lung disease	-0.0002	(-0.001–0.0009)	0.0008	(0.0005–0.002)	0.00019	(-0.0007–0.001)
Depression	-0.004	(-0.006–-0.002)	0.00004	(-0.001–0.0013)	-0.0038	(-0.005– -0.002)
Learning disability	-0.0003	(-0.002–0.001)	0.0012	(-0.000–0.002)	-0.0005	(-0.0006– 0.001)
Gastroesophageal reflux	-0.059	(-0.19– -0.009)	-0.08	(-0.002– -0.13)	-0.07	(-0.10– -0.031)
Obesity*	0.049	(0.024–0.07)	0.079	(0.024–0.13)	0.114	(0.09–0.137)
Asthma	-0.015	(-0.07–0.04)	-0.057	(-0.83–0.02)	-0.015	(-0.075–0.045)
Movement Disorder	-0.13	(-0.186– -0.07)	-0.16	(-0.24– -0.08)	-0.14	(-0.18– -0.09)
Recurrent Headaches	-0.037	(-0.016– -0.05)	-0.10	(-0.14– -0.063)	-0.025	(-0.044– -0.006)

*Diabetes (Fasting blood sugar ≥ 126), Obesity (body mass index over 30), Hypertension (systolic blood pressure ≥ 140 mm Hg and/or diastolic blood pressure ≥ 90 mm Hg).

 The results of analysis by gender showed that movement disorder, recurrent headaches and gastroesophageal reflux were significantly concentrated in the people with lower SES in both men and women. Obesity was also significantly higher in both men and women in the high SES. There was no inequality in the distribution of other studied diseases (Table 3).

**Figure 1:**
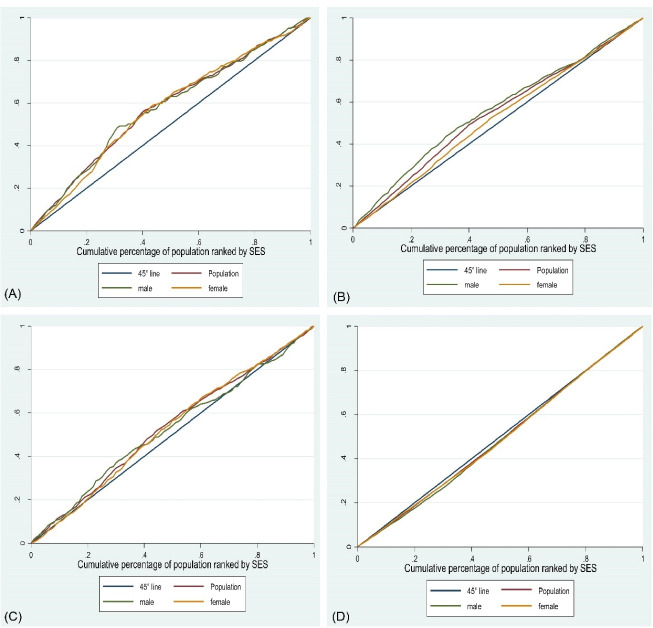
Concentration Curve of Movement Disorder **(A)**, Recurring Headache **(B)**, Gastroesophageal Reflux Disease **(C)**, and Obesity **(D)** Among Total Population, Men and Women Based on Their Socioeconomic Status.

 In addition, in the concentration curves of the movement disorder, recurrent headaches and gastroesophageal reflux, the concentration line is at the top of the equation line. This indicates that the disease is concentrated in individuals with low SES (Figure 1). The obesity concentration line is also located at the bottom of the equation line in the concentration curve, which indicates that the concentration of this disease is in people with high SES (Figure 1).

 Finally, the results of logistic regression analysis showed that increasing SES has a protective role against recurrent headaches and movement disorder, and is a risk factor for obesity. For example, high SES has a protective role against recurrent headaches (odds ratio [OR]: 0.61, 95% CI: 0.53, 0.7) and movement disorder (OR: 0.38, 95% CI: 0.0.29, 0.51), and is a risk factor for obesity (OR: 1.75, 95% CI: 1.49, 2.07) (Table 4).

**Table 4:** Odds Ratio for Non-communicable Diseases in Kharameh, Southwestern Iran, 2020

**Variable**		Obesity*		**Movement Disorder**		**Recurring Headaches**		**Gastroesophageal Reflux**	
		**OR (95%CI)**	**OR**_adj_ ** (95%CI)**	**OR (95%CI)**	**OR**_adj_ ** (95%CI)**	**OR (95%CI)**	**OR**_adj_ ** (95%ci)**	**OR (95%CI)**	**OR**_adj_ ** (95%CI)**
SES level	Low	0.99 (0.84–1.16)	1.11 (0.94–1.31)	0.75 (0.59–0.94)	0.76 (0.606–0.95)	0.86 (0.76–0.98)	0.93 (0.82–1.06)	1.07 (0.86–1.32)	1.07 (0.86–1.32)
	Medium	1.10 (0.94–1.29)	1.36 (1.16–1.6)	0.43 (0.33–0.67)	0.44 (0.34–0.58)	0.64 (0.56–0.73)	0.73 (0.63–0.82)	0.77 (0.62–0.97)	0.77 (0.62–0.97)
	High	1.11 (0.95–1.31)	1.48 (1.26–1.75)	0.52 (0.41–0.67)	0.54 (0.42–0.71)	0.55 (0.48–0.63)	0.65 (0.56–0.74)	0.74 (0.58–0.92)	0.73 (0.63–1.92)
	Very high	1.19 (1.02–1.4)	1.75 (1.49–2.07)	0.36 (0.28–0.48)	0.38 (0.29–0.51)	0.49 (0.42–0.56)	0.61 (0.53–0.7)	0.8 (0.64–1.01)	0.79 (0.63–1.007)
Age	30–50	1	1	1	1	1	1	1	1
	50–70	1.21 (1.01–1.23	0.98 (0.88–1.09)	1.22 (1.35–1.44)	1.16 (0.98–1.37)	1.21 (1.18–1.33)	1.09 (0.99–1.19)	0.92 (0.79–1.06)	0.7 (0.78–1.04)
Gender	Male	1	1	1	1	1	1	1	1
	Female	4.24 (3.75–4.79)	4.6 (4.07–5.22)	1.32 (1.11–1.56)	1.14 (0.96–1.36)	2.63 (2.3–2.8)	2.44 (2.2–2.69)	1.007 (0.87–1.16)	-

OR_adj_: Adjusted for age and gender. *P*-value < 0.001 was observed for all associations and was considered significant. * Obesity (body mass index over 30).

## Discussion

 The present study was performed to investigate the unequal distribution of chronic diseases in individuals with different SES in Kharameh in the year 2020. Recurrent headaches (25.8%) and hypertension (23.5%) were the most common chronic diseases in the population under study. The results of our study showed that there is a significant difference in movement disorder, recurrent headaches, gastroesophageal reflux diseases and obesity distribution in different SES population groups. These results are consistent with many other studies conducted in this field.^5,7,14,21,22^

 The results of our study showed that recurrent headaches, movement disorders, and gastroesophageal reflux disease were more concentrated among the individual with low SES compared to the high SES for both men and women. Amongchronic diseases in our study, people with movement disorders had a higher concentration index and a greater distance from the equality line. Although in our study, the level of significance is very close to significant and narrow, considering the fact that these results have been seen in other studies, we can also interpret the results in a meaningful way.^12,21^ Of course, this may be due to the fact that their illness has caused their poverty and this issue should be investigated in other studies with other methods. The results of our study on gastroesophageal reflux disease were inconsistent with a study conducted in Fasa city in Iran, which showed that reflux disease is concentrated among people with low SES.^21^

 Our results also showed that obesity is more concentrated in people with low SES in both men and women. The results of our study were consistent with many studies conducted in this regard.^1,12,23,24^ Given the change in lifestyle, lack of physical activity and the increasing use of fast foods, which are more common among people with low SES, it is also expected that the prevalence of obesity will be higher in this population group.

 In our study, there was no difference in the distribution of diabetes and hypertension among the individuals with high and low levels of the SES; however, the results of some other studies are inconsistent with ours. Emamian and colleagues showed that hypertension was higher among people with low SES, but did not see any difference in the distribution of diabetes.^25^ Numerous studies have also shown a higher concentration of hypertension among rich people.^11,23,24,26^

 In the case of diabetes, some studies have reported higher concentrations of the disease among people with low SES^1,10^ and others study reported it among people with high SES.^24,26^ The reason for the difference in the results of different studies may be that many people with low SES cannot afford to be visited by a physician because of high costs. Therefore, their disease is not diagnosed and this issue makes a difference in the distribution of the disease among high SES and low SES people, while in Iran, according to the screening and free periodic care system for diabetes and hypertension in health centers, almost all patients are identified and there is not a difference in diagnosis between people with low and high SES.

 It should be noted that SES inequalities are an important challenge in public health and health policies. In addition, inequalities are usually associated with many problems for the people with low SES, which indicates the existence of social injustice. For this reason, strategic plans are needed to reduce poverty and improve public health in people with different SES, and more attention must be paid to the access of populations with lower SES levels to health services.

 One of the limitations of our study was that we used only a population of individuals aged 35 to 70 years; therefore, more extensive studies should be performed on all age groups. In addition, our study was cross-sectional which may not show the temporal relationship between low SES and disease well enough. Despite these limitations, our study was conducted on a large sample size of baseline data from a cohort study. It should also be noted that so far, very few studies have been conducted on the SES inequalities in headache diseases, movement disorders and gastroesophageal reflux.

 In conclusions, non-communicable diseases have a significantly higher concentration among people of lower SES. Given the increasing trend of chronic diseases and the observed health inequalities in their distribution, policymakers should be encouraged to provide equal access to healthcare for all people, particularly in low- and middle-income settings.
